# Strength-Endurance: Interaction Between Force-Velocity Condition and Power Output

**DOI:** 10.3389/fphys.2020.576725

**Published:** 2020-10-09

**Authors:** Jean Romain Rivière, Nicolas Peyrot, Matthew R. Cross, Laurent A. Messonnier, Pierre Samozino

**Affiliations:** ^1^Univ Savoie Mont Blanc, Laboratoire Interuniversitaire de Biologie de la Motricité, EA 7424, Chambéry, France; ^2^Le Mans Université, Movement-Interactions-Performance, MIP, EA 4334, Le Mans, France

**Keywords:** force-velocity relationship, power-velocity relationship, test to exhaustion, repeated jump test, power reserve, force-velocity ratio, power-velocity-endurance profile

## Abstract

**Context:**

Strength-endurance mainly depends on the power output, which is often expressed relative to the individual’s maximal power capability (*P*_max_). However, an individual can develop the same power, but in different combinations of force and velocity (force-velocity condition). Also, at matched power output, changing the force-velocity condition results in a change of the velocity-specific relative power (*P*_max_*v*), associated with a change in the power reserve. So far, the effect of these changing conditions on strength-endurance remains unclear.

**Purpose:**

We aimed to test the effects of force-velocity condition and power output on strength-endurance.

**Methods:**

Fourteen sportsmen performed (i) force- and power-velocity relationships evaluation in squat jumps and (ii) strength-endurance evaluations during repeated squat jump tests in 10 different force-velocity-power conditions, individualized based on the force- and power-velocity relationships. Each condition was characterized by different (i) relative power (%*P*_max_), (ii) velocity-specific relative power (%*P*_max_*v*), and (iii) ratio between force and velocity (*R*_Fv_). Strength-endurance was assessed by the maximum repetitions (*SJ*_Rep_), and the cumulated mechanical work (*W*_tot_) performed until exhaustion during repeated squat jump tests. Intra and inter-day reliability of *SJ*_Rep_ were tested in one of the 10 conditions. The effects of %*P*_max_, %*P*_max_*v*, and *R*_Fv_ on *SJ*_Rep_ and *W*_tot_ were tested via stepwise multiple linear regressions and two-way ANOVAs.

**Results:**

*SJ*_Rep_ exhibited almost perfect intra- and inter-day reliability (ICC=0.94 and 0.92, respectively). *SJ*_Rep_ and *W*_tot_ were influenced by %*P*_max_*v* and *R*_Fv_ (*R*^2^ = 0.975 and 0.971; RSME=0.243 and 0.234, respectively; both *p* < 0.001), with the effect of *R*_Fv_ increasing with decreasing %*P*_max_*v* (interaction effect, *p* = 0.03). %*P*_max_ was not considered as a significant predictor of strength-endurance by the multiple regressions analysis. *SJ*_Rep_ and *W*_tot_ were higher at lower %*P*_max_*v* and in low force-high velocity conditions (i.e., lower *R*_Fv_).

**Conclusion:**

Strength-endurance was almost fully dependent on the position of the exercise conditions relative to the individual force-velocity and power-velocity relationships (characterized by %*P*_max_*v* and *R*_Fv_). Thus, the standardization of the force-velocity condition and the velocity-specific relative power should not be overlooked for strength-endurance testing and training, but also when setting fatiguing protocols.

## Introduction

Repetitive near-maximal- or maximal-intensity efforts, such as sprinting, rowing, jumping, or stair climbing, are frequent in daily life and sporting activity. The key to successful performance during repeated movements relies on the production of mechanical power and its maintenance over a series of repetitions until task completion.

Power production capabilities depend on movement velocity and are well-represented by the parabolic power-velocity (*P-v*) relationship during multi-joint movements (Bobbert, 2012; Samozino et al., 2012; Jaric, 2015). The apex of the *P-v* relationships corresponds to the maximal power attained at optimal velocity (*P*_max_), which is commonly accepted as a macroscopic measure of dynamic strength capabilities (Jaric, 2015; Alcazar et al., 2017). The ability to maintain power over a series of movements (i.e., strength-endurance) depends primarily on the output magnitude and is well-illustrated by the power-time relationship. Two distinct power-time relationships have been reported to characterize strength-endurance: (i) the inverse hyperbolic relationship between the absolute or relative power output and the duration during which this given power can be maintained, which can be obtained from 3 to 5 tests to exhaustion (Monod and Scherrer, 1965; Burnley and Jones, 2016), and (ii) the decrease in instantaneous power output over time during a single all-out exercise, which is instead associated with fatigability indices, such as the rate of power output loss over 30-s all-out cycling (Bar-Or, 1987). However, the same absolute or relative-to-*P*_max_ (%*P*_max_) power output can be developed in high force-low velocity conditions or in low force-high velocity conditions, and these different force-velocity (*F-v*) conditions can be interpreted as distinct ratios between the force output and the movement velocity (*R*_Fv_).

The effect of *R*_Fv_ on strength-endurance has been studied indirectly by investigating the effect of movement velocity using cyclic (e.g., cycling) and acyclic movements (e.g., knee extension; Elert and Gerdle, 1989; Barker et al., 2006). Due to the specificity of cyclic movements, velocity is indirectly controlled by adjusting movement frequency (e.g., the pedaling cadence) or using a specific set-ups (Dorel et al., 2003; Tomas et al., 2010). During all-out exercises, higher fatigability has been systematically observed at higher compared to lower movement frequencies in cyclic movements (i.e., cycling; e.g., Sargeant et al., 1981; Beelen and Sargeant, 1991a). However, there is little consensus in acyclic movements (i.e., knee extension and shoulder flexion) since some studies report higher fatigability at higher movement velocities (e.g., Mathiassen, 1989; Morel et al., 2015) while others report opposite results (Elert and Gerdle, 1989; Dalton et al., 2012). Moreover, *R*_Fv_ and %*P*_max_ conditions were not fixed at each repetition over the tests due to the decrease in power output throughout all-out exercises. Consequently, it is challenging to evaluate the effects of *R*_Fv_ and %*P*_max_ on strength-endurance, as well as the interactions between both mechanical conditions by the mean of all-out exercises. During tests to exhaustion performed at constant power output, only cyclic movements (i.e., cycling and paddling) were used to study the effect of *R*_Fv_ on strength-endurance. Similarly, there is a lack of consensus since some studies reported lower strength-endurance at higher movement frequencies (Carnevale and Gaesser, 1991; Barker et al., 2006) and others, lower strength-endurance at lower movement frequencies (Leveque et al., 2002; Bessot et al., 2006). Moreover, the sole effect of *R*_Fv_ cannot be examined when using cyclic movements due to the concomitant influence of both movement frequency and velocity on strength-endurance. Indeed, movement frequency alone impacts strength-endurance by changing (i) rest between repetitions and (ii) contraction number during a test of fixed duration (Enoka and Stuart, 1992; Broxterman et al., 2014). A lower time-to-exhaustion observed at higher movement frequencies can thus be due to shorter rest time between contractions and/or more contractions and/or higher contraction velocities. Overall, investigating the effect *R*_Fv_ on strength-endurance requires (i) the use of an acyclic movement, allowing the dissociation with the effect of movement frequency, and (ii) the use of time-to exhaustion at constant power to control force-velocity and power output conditions throughout the test.

In parallel to *R*_Fv_, strength-endurance can also be influenced by the power reserve. Indeed, due to the parabolic shape of the *P-v* relationship, a change in *R*_Fv_ at a matched %*P*_max_ is associated with a change in the power reserve. This reserve corresponds to the difference between the maximal power capability at a specific velocity and the power output at the same specific velocity (Sargeant, 1994, 2007; Zoladz et al., 2000). This reserve can also be interpreted as a velocity-specific relative power (%*P*_max_*v*): the lower %*P*_max_*v*, the larger the power reserve. When considering the same %*P*_max_, low force-high velocity conditions (often close to the optimal velocity) are associated with larger power reserve and lower %*P*_max_*v*, and might improve strength-endurance (Sargeant, 1994, 2007; Zoladz et al., 2000). Nevertheless, due to the concomitant change of *R*_Fv_ and %*P*_max_*v* at matched %*P*_max_, it remains unclear whether the influence of *R*_Fv_ on strength-endurance is independent of %*P*_max_*v*. Also, as matched %*P*_max_*v* can lead to different %*P*_max_, the question of which of the two indices better represents exercise intensity remains unanswered.

Clarifying the effect of %*P*_max_, %*P*_max_*v*, and *R*_Fv_ on strength-endurance could be of great interest for scientific and training purposes since typical strength-endurance evaluations have been standardized across individuals based on (i) the same relative load (e.g., percentage of the one-repetition maximum; Mayhew et al., 1992), (ii) the same movement velocity across individuals (Câmara et al., 2012) or (iii) the same resistive force per bodyweight during all-out cycling exercises (Bar-Or, 1987). Thus, provided *R*_Fv_ affects performance, inter-individual differences in strength-endurance observed with these commonly used methods could be mainly due to different mechanical conditions relative to individual capabilities (i.e., %*P*_max, %_*P*_max_*v*, and/or *R*_Fv_) rather than different physical abilities. These methods could represent both an inaccurate and non-specific means of assessing strength-endurance, and lead to practically ineffective testing and training regimes.

The aim of the present study was to test the effects of force-velocity condition (i.e., *R*_Fv_) and power output (i.e., %*P*_max_ and %*P*_max_*v*) on strength-endurance using an acyclic movement. We hypothesized that decreasing velocity-specific relative power (%*P*_max_*v*) increases strength-endurance *via* increasing power reserve, even if it led to no change or an increase in %*P*_max_. We theorized that *R*_Fv_ influences strength-endurance independently from %*P*_max_*v*, due to the likely different etiology of fatigue between high force-low velocity and low force-high velocity conditions (Enoka and Stuart, 1992; Morel et al., 2015, 2019).

## Materials and Methods

### Participants

Fourteen healthy participants (12 males and 2 females, age = 20 ± 2 years, mass = 73 ± 7 kg and height = 1.79 ± 0.09 m) gave their written informed consent to participate in this study, with all procedures in agreement with the declaration of Helsinki and the ethical standards of a local committee. All were involved in regular physical activity (14 ± 7 h of training per week) and were accustomed to strength-based resistance training (i.e., habitual use of submaximal to maximal loads). All participants were free of musculoskeletal pain or injury during the study.

### Design

The main limitations of previous works were addressed in this study by using jumping exercises due to (i) the possibility to dissociate rest between repetitions from movement velocity, (ii) the acute and reliable quantification of the mean force, velocity, and power output by lower limbs, and (iii) its similarity to typical iso-inertial movements observed in sport and testing batteries.

**FIGURE 1:**
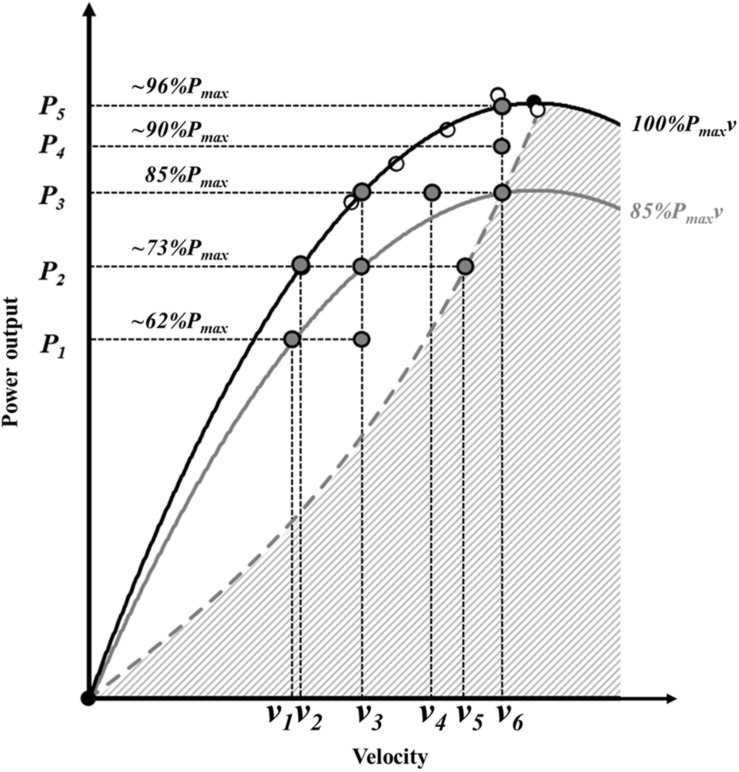
Typical individual power-velocity relationship representing 100 (black curve) and 85%*P*_max_*v* (gray curve), associated with single maximal squat jumps in different loading conditions (white points) and the 10 *F-v-P* conditions (gray points). Each *F-v-P* conditions is defined by specific power and velocity coordinates. The dashed gray curve represents the different power-velocity conditions for jumps without load, from sub-maximal to maximal jump height. The crosshatched area under the gray and the black curve represents all power-velocity conditions that require assistance (i.e., total load lower than body mass), and thus were not measured.

To test the effects of %*P*_max_*v, %P*_max_, and *R*_Fv_ on strength-endurance, repeated squat jumps (RSJ) tests to exhaustion were performed in various force-velocity-power (*F-v-P*) conditions. Overall, 10 *F-v-P* conditions were determined relative to individual *P-v* relationship (detailed in the following sections), which meant conditions were graphically positioned on or under the *P-v* curve (gray points, Figure 1). This positioning of the *F-v-P* conditions implies each condition has similar coordinates relative to individual maximal capabilities (i.e., the *P-v* relationship), but various individual absolute force, velocity and power values. Thus, each *F-v-P* conditions was characterized by different power output (*P*_1_ to *P*_5_) and velocity (*v*_1_ to *v*_6_), expressed relative to the individual *P-v* relationship (Figure 1). In addition, the positioning of *F-v-P* conditions follows the constraint imposed by dynamics principles during a vertical jump with and without additional load (represented by the white area under the *P-v* curve and the dashed gray line, respectively, Figure 1). The remaining crosshatched area represents *F-v-P* conditions requiring a simulated reduction in body weight with assistance.

The 10 *F-v-P* conditions were selected to represent: (i) 3 velocity conditions at two %*P*_max_ (corresponding to *P_3_v_3_*, *P_3_v_4_*, and *P_3_v_6_* at 85%*P*_max_ and to *P_2_v_2_*, *P_2_v_3_*, and *P_2_v_5_* at ∼73%*P*_max_), (ii) 3 velocity conditions at two %*P*_max_*v* (corresponding to *P_1_v_1_*, *P_2_v_3_*, and *P_3_v_6_* at 85%*P*_max_*v* and *P_2_v_2_*, *P_3_v_3_*, and *P_4_v_6_* at 100%*P*_max_*v*) and (iii) 3 power conditions at two velocities (corresponding to *P_5_v_6_*, *P_4_v_6_*, and *P_3_v_6_* at *v*_6_ and to *P_3_v_3_*, *P_2_v_3_*, and *P_1_v_3_* at *v*_3_). Note that all *F-v-P* conditions were determined only using power and velocity values to graphically position them relative to power capability as a common reference (i.e., *P-v* relationship), but changes in velocity across all different power conditions correspond also to changes in *R*_Fv_.

### Protocol

**FIGURE 2:**
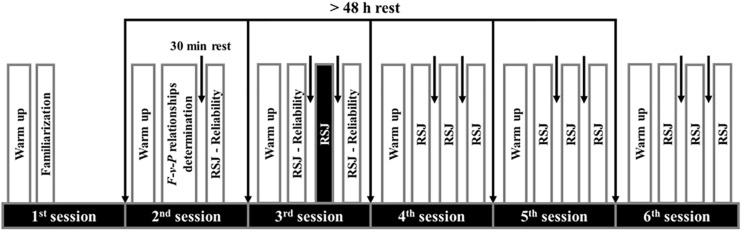
Schematic overview of the experimental design realized by each participant.

This study comprised six sessions, separated by more than 48-h of rest (Figure 2). The first session familiarized participants in performing (i) single maximal effort squat jump (SJ) with and without load (range of loads detailed in Section “Force- and Power-Velocity Relationships Assessment”) and (ii) unloaded RSJs test until exhaustion (see section “Measurements and Data Analysis” for the exact definition of exhaustion). In the second session, individual *F-v* and *P-v* relationships of the lower limbs were evaluated from SJ with and without additional loads, then RSJ test was performed in one specific *F-v-P* conditions (*P_3_v_4_*) for inter-day reliability analysis. From the third to the sixth session, each participant performed 12 RSJ tests randomly organized into 3 per session and separated by 30 min of passive rest (e.g., Karsten et al., 2016; Triska et al., 2017), which corresponded to: 1 RSJ test in each of the 10 *F-v-P* conditions, 1 RSJ test repeated one more time to assess intra-day reliability in the specific *F-v-P* conditions (*P_3_v_4_*) and 1 RSJ test was not included in the data analysis of the present study (black vertical RSJ bar, Figure 2), because this test is dedicated to answer another aim not addressed here.

The six sessions began with body mass measurements and a standardized warm-up consisting of 5 min of self-paced treadmill running followed by ∼15 min of dynamic lower-limb movements (including unloaded squats with maximal intention and sub-maximal and maximal SJs in unloaded and loaded conditions) and concluding with 5 min of non-fatiguing personally selected exercises.

### Familiarization Sessions

**FIGURE 3:**
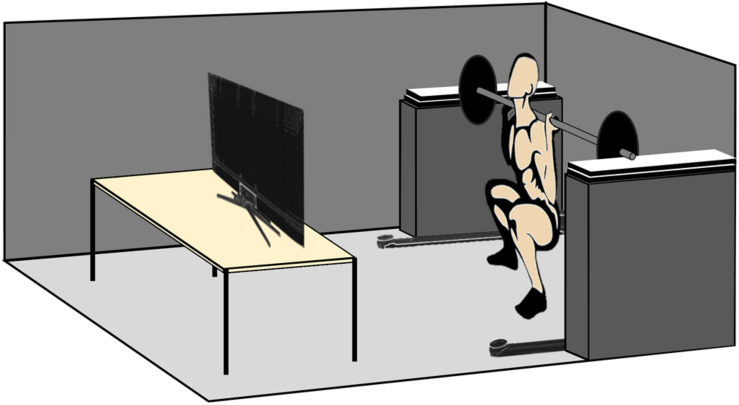
Schematic setup for all squat jumps performed to determine individual *F-v* and *P-v* relationships and during RSJ tests to exhaustion.

During the first session, the familiarization occurred in two distinct sets. The first set aimed to familiarize participants with the *F-v* and *P-v* relationships evaluation procedures. This first set included the same procedures as during the session 2, which are described in the next section, “Force- and Power-Velocity Relationships Assessment.” The second set aimed to familiarize participants with the RSJ test. This second set comprised (i) three trials of unloaded RSJ, targeting ∼50% of maximal jump height, separated each by 5 min of rest and ended when 10 successive repetitions were successfully performed at the target and, after 30 min of passive rest, (ii) two unloaded RSJ tests, aiming for maintaining the effort of maximal jump height until exhaustion, interspersed by 30 min of passive rest. During this familiarization session, individual starting position for RSJ tests and *F-v* and *P-v* relationships assessment was recorded and was standardized throughout the study. The preferential starting position was chosen by the participant, which has been shown as the method with which force, velocity, and power output are maximized and most reliable in squat jump (Janicijevic D. et al., 2019; Janicijevic D. N. et al., 2019). Using a barbell or a wooden dowel held across the shoulders, the starting position was matched with lateral adjustable supports (∼1 cm resolution), preventing participants from going beyond the starting position during the downward movement of SJ (Figure 3). Individual push-off distance (*h*_*po*_) was determined as the difference between the length of the lower limbs extended with maximal foot plantarflexion (iliac crest-toe distance) and the vertical distance between iliac crest and ground in the starting position.

### Force- and Power-Velocity Relationships Assessment

The determination of individual *F-v* and *P-v* relationships included 5 SJs with loading conditions ranging from 0 to 100% of body weight, with each condition performed twice. For each trial, participants stood stationary holding a barbell on their shoulders for additional-load conditions or a wooden dowel (∼400 g) for the unloaded condition (i.e., 0% of body weight). They lowered the bar to reach their individual starting position and after maintaining this position for 2 to 3 s, they were asked to jump maximally without countermovement. They were also prompted to touch down in the same leg position as they took off: extended leg with foot plantar flexion. If these requirements were not met, the trial was discarded, and then repeated. The trial with the greatest jump height across all trials was used to determine individual *F-v* and *P-v* relationships (Samozino et al., 2008, 2014). When the force exerted against a certain load led to the coefficient of determination of the *F-v* relationship to be lower than 0.96, a third repetition was performed with that specific load to infirm or confirm the trial.

### Repeated Jump Test

For each RSJ test, the practical setting of a given *F-v-P* condition consisted of modulating the additional load and the jump height based on fundamental laws of dynamics and following the equations proposed and validated by Samozino et al. (2008). Briefly, during the push-off phase of SJs, the mean force (*F*, Eq. 1), velocity (*v*, Eq. 2), and power (*P*, Eq. 3) developed by the lower limbs can be expressed as:

$$F=\left(m_{\text{body}}+m_{\text{bar}}\right)g\left(\frac{h}{h_{\text{po}}}+1\right)\;\left(Eq.1\right)$$

$$v=\sqrt{\frac{g\,h}{2}}\;\left(Eq.2\right)$$

$$P=\left(m_{\text{body}}+m_{\text{bar}}\right)g\left(\frac{h}{h_{\text{po}}}+1\right)\sqrt{\frac{g\,h}{2}}\;\left(Eq.3\right)$$

where *m*_body_ is the body mass, *m*_bar_ the mass of the bar (including the mass of the bar [10 kg] and the additional mass), *g* the gravitational acceleration (9.81 m.s^–2^), and *h* the jump height. From Eqs 1 and 2, the jump height (Eq. 4) and the additional mass (Eq. 5) can be computed as a function of the targeted *F-v-P* conditions:

$$h=\frac{2\,v^{2}}{g}\;\left(Eq.4\right)$$

$$m_{\text{bar}}=\frac{\frac{P}{v}}{g\,\left(\frac{h}{h_{\text{po}}}\right)+1}-m_{\text{body}}\;\left(Eq.5\right)$$

Consequently, participants were instructed to reach a targeted jump height under a specific loading condition, which allowed them to perform an RSJ test in targeted *F-v-P* conditions. The jump height was self-controlled and aided by continuous visual feedback of the jump height that was displayed, repetition by repetition, to the screen in front of the participant (Figure 3). Where the required additional mass was lower than the mass of the bar, participants wore a weighted vest with the appropriated added load (0.5 kg resolution) and the wooden dowel. The jumping frequency was adjusted at each RSJ test, considering 2.5 s rest time between two successive SJs. The jumping frequency was monitored using two audible beeps to signal (i) the initiation of the downward movement to reach the starting position and (ii) the initiation of the jump. Participants were verbally encouraged to maintain the targeted jump height as long as possible (i.e., until exhaustion). Once jump height dropped below the target, participants were provided with strong encouragements to continue with maximal intent (i.e., aiming for maximal height). All procedures were monitored by the experimenters via their own screen, hidden from the participants during their trials.

### Measurements and Data Analysis

For SJs performed during RSJ tests and *F-v* and *P-v* relationships assessment, force, velocity, and power developed during the push-off phase were computed using Eqs 1–3. The jump height was determined from fundamental laws of dynamics and aerial time (Asmussen and Bonde-Petersen, 1974), the latter being obtained using an infrared timing system (OptoJumpNext, Microgate, Bolzano, Italy). For each participant, the *F-v* and *P-v* relationships were determined from *F*, *v*, and *P* values obtained from the 5 loading SJ conditions and were used to extrapolate *F*_0_ and *v*_0_, the y and × intercept of the *F-v* relationship, respectively. Then, *P*_max_ was computed as (Samozino et al., 2012):

$$P_{m\,a\,x}=\frac{F_{0}\,v_{0}}{4}\;\left(Eq.6\right)$$

For each of the 10 RSJ conditions, *R*_Fv_ was computed as the ratio between the force developed (expressed relative to *F*_0_) and the velocity (expressed relative to *v*_0_). Exhaustion was defined as the inability to perform three consecutive jumps above 95% of the targeted jump height. Strength-endurance was quantified by (i) the maximum repetitions (*SJ*_Rep_) and (ii) the cumulated mechanical work output (*W*_tot_) associated to *SJ*_Rep_. *SJ*_Rep_ corresponded to all repetitions preceding exhaustion, excluding the three jumps below the limit of 95% of the targeted performance and *W*_tot_ was computed as the sum of the mechanical work of all repetitions of *SJ*_Rep_.

### Statistical Analysis

All data are presented as mean ± standard deviation (SD). Intra-set RSJ height variability around the targeted jump height value was assessed using a coefficient of variation. Also, absolute intra- and inter-day reliability of *SJ*_Rep_ in *P_3_v_4_* condition were assessed with the standard error of measurement (SEM; Hopkins et al., 2001) expressed in raw units and standardized to inter-individual SD. Relative intra- and inter-day reliability of *SJ*_Rep_ in *P_3_v_4_* condition were assessed with intra-class correlation coefficient (ICC), which was interpreted as almost perfect (0.81 to 1.00), substantial (0.61 to 0.80), moderate (0.41 to 0.60), fair (0.21 to 0.40), slight (0.01 to 0.20), or poor (<0.01; Landis and Koch, 1977). The difference between the two trials was tested with the paired sample *t*-test.

The respective effects of %*P*_max_, %*P*_max_*v*, and *R*_Fv_ on both *SJ*_*rep*_ and *W*_tot_ were examined using two separate stepwise multiple linear regressions performed from averaged data of the 10 *F-v-P* conditions of RSJ tests (*n* = 10), with %*P*_max_, %*P*_max_*v*, and *R*_Fv_ as independent variables and *SJ*_*rep*_ or *W*_tot_ (log-transformed to support linearity of relationships, Monod and Scherrer, 1965; Jones and Vanhatalo, 2017) as the dependent variable. To test the main effects of %*P*_max_, %*P*_max_*v* and *R*_Fv_ on both *SJ*_*rep*_ and *W*_tot_, as well as their interaction, 2 two-way ANOVAs with repeated measures were performed on *SJ*_*rep*_ and *W*_tot_, separately: (i) effects of *R*_Fv_ (low, medium and high levels) and %*P*_max_ (∼73%*P*_max_ and ∼85%*P*_max_) and (ii) effects of *R*_Fv_ (low, medium, and high levels) and %*P*_max_*v* (85%*P*_max_*v* and 100%*P*_max_*v*). Each ANOVA was performed after checking for distribution normality and equality of variance with Shapiro–Wilk’s and Mauchly’s test, respectively. In the case of non-normality and violation of the assumption of sphericity, the non-linear logarithm transformation and the Greenhouse–Geisser’s correction were applied, respectively (Sainani, 2012). Holm’s *post hoc* test was used to highlight significant differences between conditions, as well as simple main effects to test the effect of the first main factor at each level of the second factor, and *vice-versa*. For all statistical analyses, an alpha value of 0.05 was accepted as the level of significance.

## Results

All individual *F-v* relationships fitted by linear regressions showed very high quality (*R*^2^ = 0.98 to 1; *p* < 0.001), and were associated to *F*_0_ of 2202 ± 317 N (30.1 ± 3.5 N.kg^–1^), *v*_0_ of 2.79 ± 0.43 m.s^–1^, *P*_max_ of 1542 ± 329 W (21.0 ± 4.0 W.kg^–1^) and *h*_*po*_ of 0.45 ± 0.06 m. The SEM, ICC, and *t*-test’s *p*-values between the trials performed in the *P_3_v_4_* condition to assess intra-day and inter-day reliability are presented in Table 1. RSJ additional load, targeted jump height, intra-set coefficient of variation of jump height, and jumping frequency associated with the 10 *F-v-P* conditions are presented in Table 2. *SJ*_Rep_, *R*_Fv_, *W*_tot,_ as well as the targeted and achieved absolute and relative force, velocity, and power values associated with the 10 *F-v-P* conditions are presented in Table 3.

**TABLE 1:** Mean ± SD of the maximum repetitions in *P*_3_*v*_4_ condition obtained from the two trials to assess intra-day and inter-day reliability analysis.

	Maximum repetitions			Reliability		
			
	Trial 1	Trial 2	*p*-Value	SEM	Standardized SEM (inference)	ICC [95%CI]
Intra-day reliability	17.8 ± 7.6	16.9 ± 9.4	0.363	2.36	0.28 (small)	0.94 [0.83;0.98]
Inter-day reliability	17.8 ± 8.8	16.9 ± 9.4	0.480	2.79	0.31 (small)	0.92 [0.8;0.97]

**TABLE 2:** Mean ± SD of additional load, intra-set RSJ jump height coefficient of variation and jumping frequency for the 10 *F-v-P* conditions.

		Additional load (kg)	Targeted jump height (cm)	Coefficient of variation (%)	Jumping frequency (Hz)
***P*_5_**	***v*_6_**	11.2 ± 1.7	25.8 ± 5.4	1.98 ± 1.28	0.36 ± 0.04
***P*_4_**	***v*_6_**	5.9 ± 1.2	25.8 ± 5.4	2.34 ± 0.75	0.35 ± 0.02
***P*_3_**	***v*_6_**	0.2 ± 0.5	25.8 ± 5.4	3.24 ± 0.78	0.35 ± 0.01
	***v*_4_**	19.9 ± 4.3	19.5 ± 4.4	3.35 ± 1.33	0.34 ± 0.01
	***v*_3_**	46.9 ± 12.6	14.2 ± 3.7	3.39 ± 1.65	0.31 ± 0.05
***P*_2_**	***v*_5_**	0.1 ± 0.5	21.9 ± 4.8	4.58 ± 3.15	0.34 ± 0.01
	***v*_3_**	31.3 ± 9.7	14.2 ± 3.7	5.22 ± 1.86	0.33 ± 0.02
	***v*_2_**	68.8 ± 17.1	9.2 ± 2.6	6.04 ± 4.38	0.28 ± 0.05
***P*_1_**	***v*_3_**	15.6 ± 6.7	14.2 ± 3.7	5.89 ± 0.88	0.33 ± 0.02
	***v*_1_**	52.7 ± 14.3	8.7 ± 2.6	8.43 ± 3.98	0.32 ± 0.01

**P*_*i*_: power output expressed relative to the power-velocity relationship; *v*_*i*_: velocity expressed relative to the power-velocity relationship.*

**TABLE 3:** Mean ± SD of the maximum repetitions, force-velocity ratio, absolute and relative achieved and targeted force, velocity and power output for each of the 10 *F-v-P* conditions.

		Maximum repetitions	Cumulated mechanical work (J)	Power (W)		Power (%*P*_max_)		Power (%*P*_max_*v*)		Force (N)		Velocity (m.s^–1^)		Force-velocity ratio (*R*_Fv_)		Mechanical work (J)	
										
				Targeted	Achieved	Targeted	Achieved	Targeted	Achieved	Targeted	Achieved	Targeted	Achieved	Targeted	Achieved	Targeted	Achieved
***P*_5_**	***v*_6_**	6.21 ± 4.56	4079 ± 2925	1466 ± 318	1422 ± 316	95.6 ± 2.4	92.5 ± 4.4	100.0 ± 0.0	96.8 ± 3.3	1297 ± 169	1282 ± 164	1.12 ± 0.12	1.10 ± 0.13	1.49 ± 0.21	1.50 ± 0.22	587 ± 97	582 ± 103
***P*_4_**	***v*_6_**	17.00 ± 9.43	9288 ± 5381	1372 ± 298	1363 ± 294	89.4 ± 1.9	88.8 ± 2.6	93.6 ± 0.4	92.9 ± 1.0	1213 ± 158	1213 ± 157	1.12 ± 0.12	1.11 ± 0.12	1.40 ± 0.21	1.40 ± 0.21	555 ± 76	550 ± 99
***P*_3_**	***v*_6_**	58.79 ± 37.39	29702 ± 19616	1278 ± 279	1248 ± 262	85.0 ± 0.0	83.3 ± 2.0	85.0 ± 0.0	83.2 ± 2.0	1130 ± 148	1119 ± 139	1.12 ± 0.12	1.10 ± 0.12	1.30 ± 0.21	1.30 ± 0.19	512 ± 97	508 ± 89
	***v*_4_**	19.86 ± 7.47	12016 ± 5391	1278 ± 279	1270 ± 275	85.0 ± 0.0	84.9 ± 2.4	92.0 ± 1.0	91.4 ± 2.0	1295 ± 167	1296 ± 162	0.97 ± 0.11	0.97 ± 0.12	1.71 ± 0.18	1.77 ± 0.18	590 ± 56	589 ± 102
	***v*_3_**	4.21 ± 2.97	2892 ± 2099	1278 ± 279	1240 ± 247	85.0 ± 0.0	86.5 ± 3.7	100.0 ± 0.0	97.3 ± 4.2	1518 ± 201	1506 ± 188	0.83 ± 0.11	0.82 ± 0.10	2.37 ± 0.10	2.41 ± 0.12	693 ± 117	686 ± 117
***P*_2_**	***v*_5_**	148.21 ± 88.56	70858 ± 41274	1115 ± 245	1115 ± 254	72.6 ± 0.8	72.4 ± 1.8	78.3 ± 1.8	78.2 ± 2.3	1069 ± 137	1073 ± 139	1.03 ± 0.12	1.03 ± 0.12	1.34 ± 0.20	1.34 ± 0.20	490 ± 95	486 ± 87
	***v*_3_**	20.64 ± 10.97	11804 ± 6254	1115 ± 245	1112 ± 242	72.6 ± 0.8	72.4 ± 2.4	85.0 ± 0.0	84.9 ± 2.4	1324 ± 173	1324 ± 168	0.83 ± 0.11	0.83 ± 0.11	2.07 ± 0.10	2.06 ± 0.12	611 ± 57	605 ± 105
	***v*_2_**	2.93 ± 2.53	2046 ± 1777	1115 ± 245	1057 ± 252	72.6 ± 0.8	68.9 ± 7.1	100.0 ± 0.0	94.9 ± 9.4	1642 ± 222	1616 ± 219	0.67 ± 0.10	0.65 ± 0.10	3.19 ± 0.07	3.26 ± 0.20	746 ± 60	739 ± 123
***P*_1_**	***v*_3_**	124.07 ± 86.76	62586 ± 44091	951 ± 211	964 ± 218	61.9 ± 0.7	62.5 ± 1.6	74.3 ± 1.6	75.1 ± 2.4	1129 ± 145	1136 ± 144	0.83 ± 0.11	0.84 ± 0.11	1.76 ± 0.11	1.76 ± 0.11	523 ± 101	518 ± 90
	***v*_1_**	12.64 ± 6.85	8455 ± 5203	951 ± 211	969 ± 222	61.9 ± 0.7	62.9 ± 2.9	85.0 ± 0.0	86.5 ± 3.6	1444 ± 193	1453 ± 190	0.65 ± 0.10	0.66 ± 0.10	2.89 ± 0.07	2.85 ± 0.13	674 ± 104	668 ± 118

**P*_*i*_: power output expressed relative to the power-velocity relationship; *v*_*i*_: velocity expressed relative to the power-velocity relationship.*

The stepwise multiple regression analysis with *SJ*_*rep*_ as the dependent variable showed that %*P*_max_*v* (88.4% of the variance explained, beta-weight of −0.812) and *R*_Fv_ (9.1% of the variance explained, standardized beta-weight of −0.327) accounted for a significant amount of *SJ*_*rep*_ variability (*p* < 0.001; *F* = 134.187). The regression model obtained was ln(*SJ*_Rep_) = 17.042 – 0.144(%*P*_max_*v*) – 0.649(*R*_Fv_), which indicated a very high goodness of fit (*R*^2^ = 0.975, *p* < 0.001), with low residuals (RSME = 0.243).

The stepwise multiple regression analysis with *W*_tot_ as the dependent variable showed that %*P*_max_*v* (89.2% of the variance explained, beta-weight of −0.825) and *R*_Fv_ (7.9% of the variance explained, standardized beta-weight of −0.305) accounted for a significant amount of *W*_tot_ variability (*p* < 0.001; *F* = 116.866). The regression model obtained was ln(*W*_tot_) = 22.140 – 0.132(%*P*_max_*v*) – 0.545(*R*_Fv_), which indicated a very high goodness of fit (*R*^2^ = 0.971, *p* < 0.001), with low residuals (RSME = 0.234).

### Effect of R_Fv_ and %P_max_ on SJ_*rep*_ and W_tot_

**FIGURE 4:**
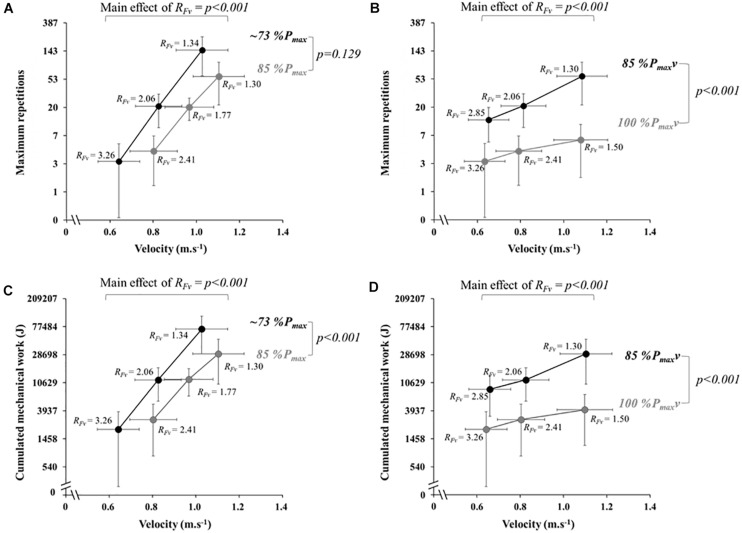
The maximum repetitions **(A,B)** and cumulated mechanical work **(C,D)**, presented on a natural logarithmic scale at different velocities, at ∼73%P_max_ (black) and 85%P_max_ (gray) on the **(A,C)**, and at 85%P_max_v (black) and 100%P_max_v (gray) on the **(B,D)**. The force-velocity ratio values associated to each condition are presented in black text. Power and force-velocity ratio main effects of the two-way ANOVAs for repeated measures are presented as black vertical and horizontal bars, respectively, with the associated *p*-values.

The two-way ANOVA with repeated measures testing the effect of %*P*_max_ and *R*_Fv_ on *SJ*_*rep*_ showed a main effect of *R*_Fv_ (*p* < 0.001) and *R*_Fv_ × %*P*_max_ interaction (*p* < 0.001), but no main effect of %*P*_max_ (*p* = 0.129; Figure 4A). *Post hoc* comparisons revealed significant differences (*p* < 0.001) for all comparisons between the three *R*_Fv_ levels, with an increase of *SJ*_*rep*_ when *R*_Fv_ decreases. A simple main effect of %*P*_max_ was observed at the highest level of *R*_Fv_ (*p* < 0.001), but not at the two lower levels (*p* = 0.129 and *p* = 0.782, for the lowest and the middle level, respectively). A simple main effect of *R*_Fv_ was observed at the two levels of power (*p* < 0.001).

The two-way ANOVA with repeated measures testing the effect of %*P*_max_ and *R*_Fv_ on *W*_tot_ showed a main effect of *R*_Fv_ (*p* < 0.001) and %*P*_max_ (*p* < 0.001), and *R*_Fv_ × *%P*_max_ interaction (*p* < 0.001*;* Figure 4C). *Post hoc* comparisons revealed significant differences for all comparisons between the three *R*_Fv_ levels (*p* < 0.001), with an increase of *W*_tot_ when *R*_Fv_ decreases. A simple main effect of %*P*_max_ was observed at the low level of *R*_Fv_ (*p* < 0.001), but not at the moderate and high levels (*p* = 0.954 et *p* = 0.323, respectively). There was a simple main effect of *R*_Fv_ at the two levels of power (*p* < 0.001).

### Effect of R_Fv_ and %P_max_v on SJ_*rep*_ and W_tot_

The two-way ANOVA with repeated measures testing the effect of %*P*_max_*v* and *R*_Fv_ on *SJ*_*rep*_ showed a main effect of *R*_Fv_ (*p* < 0.001) and %*P*_max_*v* (*p* < 0.001), and *R*_Fv_ × *P*_max_*v* interaction (*p* = 0.03; Figure 4B). *Post hoc* comparisons revealed significant differences in all comparisons between the three *R*_Fv_ levels (*p* < 0.05), with an increase of *SJ*_*rep*_ when *R*_Fv_ decreases. A simple main effect of %*P*_max_*v* was observed at each level of *R*_Fv_ (*p* < 0.001). There was a simple main effect of *R*_Fv_ at 85%*P*_max_*v* (*p* < 0.001) and a trend at 100%*P*_max_*v* (*p* = 0.078).

The two-way ANOVA with repeated measures testing the effect of %*P*_max_*v* and *R*_Fv_ on *W*_tot_ showed a main effect of *R*_Fv_ (*p* < 0.001) and %*P*_max_*v* (*p* < 0.001) and *R*_Fv_ × %*P*_max_*v* interaction (*p* < 0.001*;* Figure 4D). *Post hoc* comparisons revealed significant differences at the three *R*_Fv_ levels (*p* < 0.05), with an increase of *W*_tot_ when *R*_Fv_ decreases. A simple main effect of %*P*_max_*v* was observed at the three levels of *R*_Fv_ (*p* < 0.001). There was a simple main effect of *R*_Fv_ observed at 85%*P*_max_*v* (*p* < 0.001), but only a trend at 100%*P*_max_*v* (*p* = 0.134).

## Discussion

The main finding of this study was that strength-endurance in repeated jumping depends on force, velocity, and power conditions, expressed relative to force- and power-velocity relationships. The large intra-individual differences in both the maximum repetitions and total work produced across the 10 *F-v-P* conditions studied (from ∼3 to ∼150 repetitions and from ∼2000 to ∼70000 Joules) were almost entirely explained (∼98%) by both the velocity-specific relative power and the ratio between force and velocity to generate power. Strength-endurance was higher at lower velocity-specific relative power and in lower force-higher velocity conditions. Intra- and inter-day reliability of the RSJ test to exhaustion was acceptable and congruent with previously reported reliabilities for tests to exhaustion of approximately similar duration (e.g., Coggan and Costill, 1984; Hinckson and Hopkins, 2005).

In comparison to %*P*_max_ and *R*_Fv_, %*P*_max_*v* was the mechanical condition that affected the most strength-endurance (i.e., ∼88–89% of the variance explained in *SJ*_*rep*_ and *W*_tot_).%*P*_max_ was not a predictor of strength-endurance, notably since it does not consider the change in power capability with the force-velocity condition. Indeed, at the same %*P*_max_, the power output relative to the velocity-specific *P*_max_ (i.e., *P*_max_*v*) can be drastically different according to the force-velocity conditions and lead to substantial differences in strength endurance performance. It is worth noting that among the 10 *F-v-P* conditions, a lower %*P*_max_ was not systematically associated with a higher strength-endurance. For example, the 3 *F-v-P* conditions at ∼85%*P*_max_, ∼73%*P*_max_, and ∼62%*P*_max_ were associated with performances of ∼58, ∼21, and 12 repetitions, respectively. This further highlights the inability of %*P*_max_ to represent exercise intensity, notably when the exercises are not performed in the same force-velocity condition. Since the force-velocity condition varies during field performance and physical testing due to changing loading/resistive conditions and levers/equipment used, the common implementation of %*P*_max_ to represent exercise intensity could be challenged (e.g., Harman et al., 1987; Bundle et al., 2003). Instead, it appears that %*P*_max_*v* better represents exercise intensity, since it considers the change in the individual maximal power capabilities according to the force-velocity condition. Thus, strength endurance seems to depend primarily on power output, expressed relative to the velocity-specific maximal power, and not to the maximal power value developed at optimal velocity. This supports the importance of the power reserve (Sargeant, 1994, 2007; Zoladz et al., 2000), and in turn, the influence of maximal power capabilities (i.e., the *P-v* relationship) on the individual ability to maintain sub-maximal power over time, notably at high exercise intensities.

**FIGURE 5:**
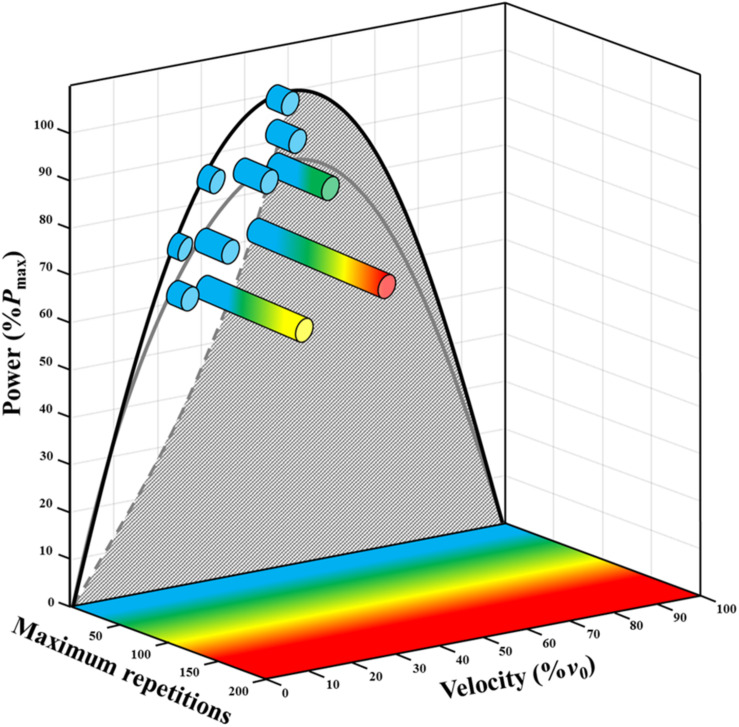
Schematic three-dimensional power-velocity-endurance relationships representing mean maximum repetitions across individuals in the 10 *F-v-P* conditions (colored horizontal cylinders). The dashed gray curve represents the different power-velocity conditions for jumps without load, from sub-maximal to maximal jump height. The crosshatched area under the gray and the black curve represents all power-velocity conditions that require assistance (i.e., total load lower than body mass), and thus were not measured.

The second strongest mechanical predictor of strength-endurance was *R*_Fv_, which explained ∼8–9% of the variance in *SJ*_Rep_ and *W*_tot_. Note that the remaining variance (∼2–3%) is likely due to measurement errors. Decreasing *R*_Fv_ (i.e., increasing movement velocity and decreasing the force output at matched %*P*_max_ or %*P*_max_*v*) resulted in increased strength-endurance. These results confirm that, when standardizing rest time between repetitions, a change in force-velocity condition influences strength-endurance independently from a change in %*P*_max_*v* (Figures 4B,D) or a change in %*P*_max_ (Figures 4A,C). These findings contrast previous hypotheses suggesting that increasing movement velocity is unbeneficial (Mathiassen, 1989; Carnevale and Gaesser, 1991; Morel et al., 2015), notably due to potentially higher proportions of fatigable type II muscle fiber recruitment (Beelen and Sargeant, 1991b; Blake and Wakeling, 2014). However, as these studies did not use standardized rest time between contractions and fixed repetitions across velocity conditions, the negative effect of low rest time in high-frequency conditions could have counteracted the positive effect of movement velocity. Additionally, as mechanical work per repetition was different across all *F-v-P* conditions, the total mechanical work produced until exhaustion is likely a better index of strength endurance, even if less practically relevant. However, although *R*_Fv_ explained a comparatively small part of the overall variance, its change led to substantial differences in strength-endurance (e.g., ∼13, ∼20, and ∼60 repetitions at 85%*P*_max_*v*, with associated *R*_Fv_ mean values of ∼2.9, ∼2.1, ∼1.3, respectively). It is worth noting that, the influence of *R*_Fv_ on strength-endurance can change according to %*P*_max_*v*, as shown by the significant *R*_Fv_ × *P*_max_*v* interaction. Indeed, the effect of *R*_Fv_ is further magnified at lower %*P*_max_*v* (Figures 4B,D). Taken together, these results show that increases in velocity and decreases in force at the same %*P*_max_*v* or %*P*_max_ during acyclic movements (e.g., repeated jumps or callisthenic exercises) are rather beneficial than detrimental and could lead to substantial change in maximum repetitions and cumulated work until exhaustion. Strength-endurance at the individual level seems to be almost fully dependent on *F-v-P* conditions, expressed relative to the individualized *F-v* and *P-v* relationships. More specifically, performance is determined by the position of the exercise mechanical conditions on or under the *F-v* and *P-v* relationships, this position being characterized by %*P*_max_*v* and *R*_Fv_ (expressed relative to *F*_0_ and *v*_0_; Figure 5).

### Limitations of the Study

One limitation of this study is the restricted range investigated relative to the entire *P-v* spectrum, and coincidentally the extrapolation of the results on the effect of %*P*_max_*v* and *R*_Fv_ on strength-endurance beyond the optimal velocity. However, the range of movement velocities explored was maximized considering conditions occurring in sports activities (i.e., inertial and resistive conditions close to bodyweight and higher). The range of power explored was also nearly maximized, from maximal jump height to jump height of ∼10 cm with different loadings. The latter was proposed as a cut-off jump height for accurate assessment of force, velocity and power output with the practical field method used in this study (García-Ramos et al., 2018). Also, although rest time between contractions was controlled in the present study, slight differences were observed in jumping frequencies across *F-v-P* conditions. However, due to the specificity of the RSJ test, other main mechanisms associated to the negative effect of movement frequency, that is, the lower effectiveness of force application (Dorel et al., 2010) and higher internal work (Zoladz et al., 2000) may have minorly affected our results. Another limitation is the focus on the understanding of the difference in strength-endurance between different *F-v-P* mechanical conditions, without considering inter-individual differences. Qualifying the physical abilities underlying differences in strength-endurance for two participants in the same %*P*_max_*v* and *R*_Fv_ would be a beneficial avenue of investigation for future research.

### Practical Applications and Perspectives for Future Studies

• Strength-endurance evaluation should be standardized according to the individual *F-v* and *P*-*v* relationships, notably *via* %*P*_max_*v* and *R*_Fv_, rather than to (i) a given percentage of maximal force (Mayhew et al., 1992), (ii) the same movement velocity across individuals (Câmara et al., 2012), or (iii) the same resistive force per bodyweight during all-out cycling exercises (Bar-Or, 1987). Without such standardization, inter-individual differences in strength-endurance could be mainly due to different %*P*_max_*v* and *R*_Fv_ conditions among individuals and not only a marker of different physical abilities. Such “Force-velocity-Power based training” could ensure strength and conditioning to improve the strength-endurance of athletes in competition-specific %*P*_max_*v* and *R*_Fv_ conditions.
• Similarly, standardizing dynamic fatiguing protocols and the subsequent fatigue assessment only relative to %*P*_max_ or maximal isometric force (Millet et al., 2011) could be challenged since each individual may experience different %*P*_max_*v* and *R*_Fv_ conditions during both phases of such experimentation. Thus, it is likely that the typical high inter-individual variability response in fatigue level (Morel et al., 2019) could be explained by the non-consideration of *F-v-P* conditions under which the evaluation or the effort was performed.

• RSJ is a reliable, practical, and modifiable method to evaluate lower limb strength-endurance in a broad range of exercise conditions specific to field situations. Indeed, the results of the present study showed that strength-endurance assessment in jumping exhibited acceptable absolute and almost perfect relative intra- and inter-day reliability. These values are in agreement with those reported in cycling for efforts of approximately similar duration (e.g., Coggan and Costill, 1984; Hinckson and Hopkins, 2005). The only requirements of an RSJ test are the measurements of body mass, push-off distance and continuous jump height over successive repetitions, and the use of Samozino et al’s validated simple method to estimate force, velocity, and power in jumping (Samozino et al., 2008; Giroux et al., 2014; Jiménez-Reyes et al., 2014; García-Ramos et al., 2019). Notably, there are many convenient methods of detecting the necessary variables (e.g., phone applications or other common devices, such as optical systems). Since different sporting scenarios involving repeated lower limb extensions feature different underlying expressions of movement frequency, force, velocity, and power output (e.g., volley-ball vs. skiing disciplines), it is possible to adapt these mechanical conditions through manipulating rest time, loading, and jump height. While the RSJ test is relatively simplistic, non-familiar cohorts of participants should be well-familiarized to ensure reasonable accuracy and reliability of assessment (Hopkins et al., 2001).

## Conclusion

Strength-endurance in jumping, either characterized as the maximum repetitions or cumulated mechanical work performed until exhaustion, depends on both the velocity-specific relative power (or the power reserve) and the underlying force-velocity condition. Strength-endurance was higher when velocity-specific relative power was lower (i.e., larger power reserve) and when the force-velocity condition to generate power was oriented toward low force-high velocity (at least until optimal velocity). The RSJ is a reliable and practical method to assess strength-endurance of the lower limbs, with the possibility to easily set these mechanical conditions, by manipulating jump height, loading and rest time between jumps. Strength-endurance in acyclic movements depends on the position of the exercise mechanical conditions, in terms of relative force, velocity and power, which can be situated on or under the force-velocity and power-velocity relationships. Since maximal capabilities (i.e., force- and power-velocity relationships) and the exercise mechanical conditions (i.e., force-velocity condition and velocity-specific relative power) influence strength-endurance performances, both should be controlled and targeted to standardize testing and training between individuals and to explore underlying mechanisms of fatigue.

## Data Availability Statement

The raw data supporting the conclusions of this article will be made available by the authors, without undue reservation.

## Ethics Statement

The studies involving human participants were reviewed and approved by Comité d’Ethique de la Recherche à l’Université Savoie Mont Blanc. The patients/participants provided their written informed consent to participate in this study.

## Author Contributions

JR, PS, NP, and LM conceived and designed the experimentation. JR conducted the experiments and wrote the manuscript. JR, MC, and PS analyzed the data. All authors read and approved the manuscript.

## Conflict of Interest

The authors declare that the research was conducted in the absence of any commercial or financial relationships that could be construed as a potential conflict of interest.
